# Spatial patterns of fat within the deep multifidus as a biomarker for chronic low back pain

**DOI:** 10.1016/j.spinee.2025.07.022

**Published:** 2025-07-05

**Authors:** Karim Khattab, Lucas K. Dziesinski, Jessica Ornowski, Jiamin Zhou, Noah B. Bonnheim, Rebecca Crawford, Aaron Scheffler, Aaron J. Fields, Conor W. O’Neill, Jeffrey C. Lotz, Jeannie F. Bailey, The REACH Investigators

**Affiliations:** a Department of Orthopedic Surgery, University of California, San Francisco, CA, USA; b Eugalyptus GmbH, Baar, Switzerland; c Department of Epidemiology and Biostatistics, University of California, San Francisco, CA, USA

**Keywords:** Chronic low back pain, Multifidus, Fat infiltration, Muscle quality, Biomarker, Spatial distribution

## Abstract

**BACKGROUND CONTEXT:**

Chronic low back pain (cLBP) patients often have elevated fat infiltration (FI) in the multifidus (MF), but it is unclear how this relates to pain and degenerative spine features. Most prior work assess MF degeneration as the average whole-muscle fat content even though deep and superficial fascicles of the MF have different structural and functional characteristics. Assessing the spatial distribution of MF FI may provide regional context for causal mechanisms which may have distinct regional presentations within the muscle.

**PURPOSE:**

This study assesses spatial patterns of MF FI at each lumbar level to identify regional differences associated with cLBP symptoms and degenerative spine features.

**STUDY DESIGN:**

This is an observational cross-sectional study.

**PATIENT SAMPLE:**

Our study sample consisted of 230 cLBP patients from the BACPAC comeback cohort who reported low back pain that has persisted for the past 3 months.

**OUTCOME MEASURES:**

Measures included MF fat-maps—a FI curve showing the spatial distribution of fat moving radially through the MF—created at each lumbar level (L1L2-L5S1). Other measures included average fat fraction in the deepest 15% of the MF (deep15 FI%), average whole-muscle fat fraction (overall FI%) and the Pain, Enjoyment of Life, and General Activity (PEG) survey score.

**METHODS:**

We collected 3T MRI and used advanced sequences (IDEAL) to map the spatial distribution of MF FI at each lumbar level. We used statistical parametric mapping to identify spatial patterns of fat in the MF associated with age, sex, and BMI. Then, we tested for differences in spatial patterns of MF FI associated with pain and adjacent disc degeneration. Next, we calculated the fat fraction in a region of interest in the deepest 15% of the MF (deep15 FI%) and used linear mixed effects modeling to compare ho w age, sex, BMI, pain, and degenerative spine features associate with the deep15 FI% and the overall FI% separately. Lastly, we used linear regression models of PEG to compare FI measures as predictors for pain.

**RESULTS:**

Elevated FI associated with PEG and adjacent disc degeneration only within the deepest 10% (p<.05) and deepest 25% (p<.01) of the MF respectively at the lower lumbar levels. Associations between demographic factors and FI were not specific to the deep MF. Older age and female sex associated with elevated FI throughout the muscle (p<.001) while higher BMI associated with elevated FI in only the superficial 60% of the MF (p<.001). Lastly, higher mean deep15 FI% but not mean overall FI% at the lower lumbar levels associated with higher PEG (p=.023).

**CONCLUSIONS:**

FI in deep regions of the MF at L4L5 and L5S1 is more strongly associated with pain and adjacent disc degeneration and less associated with age, sex, and BMI than overall FI%. We identify regional differences in MF FI related to pain which improves our understanding of cLBP-related MF degeneration and provides additional context for possible causal mechanisms of FI. Further, the “deep15” FI% is a novel MF FI measure, and possible biomarker, more strongly associated with cLBP than summary measures.

## Introduction

Most patients with chronic low back pain (cLBP) never receive a specific diagnosis and, consequently, treatment approaches are often trial-and-error [1]. One theory is that, in some patients, cLPB arises from tissue overloading secondary to spinal biomechanical instability [2–4]. It is generally recognized that biomechanical instability can come from degeneration of the passive (discs, vertebrae, facets) and active (paraspinal muscles; PSM) stabilizers [5]. Yet, degenerative features observed on clinical imaging inconsistently associate with cLBP [6].

The multifidus (MF) is a deep paraspinal muscle and uniquely spans each vertebral segment and thereby serves an important role as an active spinal stabilizer. The MF is composed of short and long (often described as deep and superficial, respectively) fascicles that are structurally and functionally unique [7–9]. Many investigations have noted associations between cLBP and MF dysfunction and degeneration [10,11] but the underlying mechanisms of MF degeneration and its causal relationship with cLBP symptoms remains poorly understood. Most prior work assess MF degeneration as the average whole-muscle fat content even though the deep and superficial MF regions have different structural and functional characteristics.

There are several proposed mechanisms of MF fat infiltration (FI) including denervation, arthrogenic inhibition, inflammation, and disuse. These mechanisms may have distinct regional presentations of MF FI [12]. Additionally, factors like age, sex, and BMI can associate with whole body FI in skeletal muscle [13] including the PSM [14], which may cloud the specific relationship between MF muscle function and cLBP. A prior study identified differences in MF FI between the deep and superficial muscle regions that associated with degenerative features of the passive spinal tissues and pain, but did not control for age, sex, and BMI [15]. Separately, a recent study reported that PSM FI patterns associate with age, sex and BMI [14], but it is still unknown whether these FI patterns are relevant to cLBP independent of these factors. It is also unknown whether an integrated analysis of both passive and active stabilizers better explains pain trends in cLBP patients.

We hypothesized that elevated FI in the deeper regions of the MF associates with degeneration of the passive spinal tissues and pain, and is less related to age, sex, and BMI than overall MF FI. To test this hypothesis, we mapped the spatial distribution of MF FI at each lumbar vertebral level in a cohort of 230 cLBP patients and assessed the association between spatial patterns of MF FI and age, sex, and BMI. Next, accounting for age, sex, and BMI, we tested for differences in spatial patterns of MF FI associated with pain and adjacent spinal degeneration. We expect that this work will lead to the development of improved muscle quality biomarkers relevant to the cLBP patient population with important implications in precision treatment.

## Methods

### Data collection and recruitment criteria

Patients were selected from the BACPAC Consortium comeback cohort [16] which, with IRB approval and informed consent, recruited 450 cLBP patients above the age of 18 who reported experiencing low back pain for more than 3 months. Exclusion criteria for enrollment included pregnancy, cancer, spine infection, ankylosing spondylitis, rheumatoid arthritis, polymyalgia rheumatica, psoriatic arthritis, lupus, vertebral fracture and cauda equina syndrome. From the BACPAC comeback cohort, we identified all 230 patients with complete imaging and survey data. From each patient we collected 3T MRI as well as demographic data and pain scores collected just prior to the scan. Pain intensity and interference was measured using the Pain, Enjoyment of Life, and General Activity (PEG) survey score [17].

### MRI scans, pathology scoring, and muscle segmentation

Lumbar scans were performed on a 3T scanner using standard clinical T1- and T2- weighted MRI sequences and advanced sequences to identify spine features and assess disc, endplate, and muscle quality. FI was measured using a 3D spoiled gradient-recalled echo (SPGR) sequence with 6 echoes and iterative decomposition of water and fat with echo asymmetry and least-squares estimation (IDEAL) [18]. Sequence specifications are presented in prior publication [18,19]. For all patients, a musculoskeletal radiologist assessed endplate defects, Modic changes, disc degeneration, facet joint osteoarthritis, nerve root contact, and central canal stenosis using T1 and T2 standard clinical sequences according to a comprehensive research-grade scoring template [19]. Disc degeneration was scored using Pfirrmann Grade. Endplate defects were measured based on presence or absence of osteoporotic fractures, Scheuermann variant, or degenerative changes. Nerve root contact was graded by the presence or absence of nerve root contact with no distinction between unilateral or bilateral contact. Facet joint osteoarthritis was identified by the presence or absence of small to large osteophytes, hypertrophy, or any narrowing of the joint space. Central canal stenosis was measured based on the presence or absence of thecal sac constriction with a loss of cerebrospinal fluid. At each disc level from L1L2 to L5S1, the right and left MF was manually segmented across 2 slices at the center of the disc. We used a combination of T1- and T2-axial images to segment the muscle, then validated the segmentations before transferring them to IDEAL images to create MF fat-maps (see [18] for full description of muscle segmentation and [15] for image sequence specifications).

### Fat-mapping of the MF

**Fig. 1.**
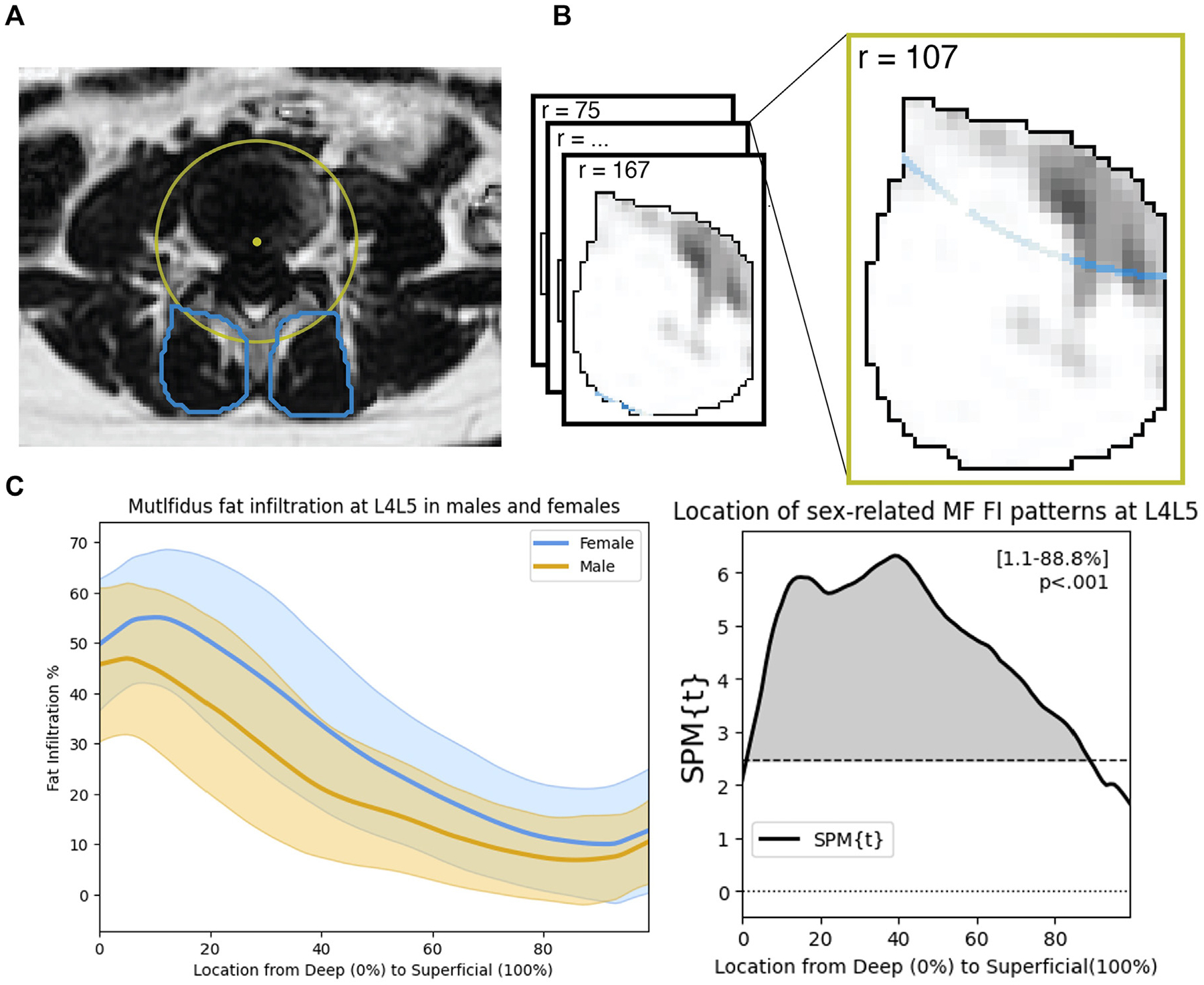
Workflow for multifidus fat-map creation and SPM analysis A) Circular regions of interest, centered at the vertebral center of rotation, are defined in the muscle segmentations at increments of 2 pixels. B) For each circular ROI, the average fat infiltration across all pixels is in the ROI is averaged. C) The fat infiltration at each ROI is plotted and distance normalized to 0% to 100% of the muscle length, resulting in a fat map curve, allowing for comparisons. ex. Fat infiltration curves for males and females at the L4L5 vertebral level D) SPM statistical tests are used to identify regions of significance (clusters) in the distribution. A region is considered significant if the SPM test statistic (black line) crosses the critical threshold (dotted line) Ex. Females have higher FI than males at L4L5 from 1.1%−88.8% of the distribution.

To investigate spatial patterns of MF FI, we used a novel fat-mapping method [15] depicted in Fig. 1. At the center of each disc, we approximated the center of rotation of the motion segment at the center point of the posterior quarter of the mid-sagittal AP diameter of the IVD (12.5% from posterior edge of disc) [20]. We defined multiple circular regions of interest (ROI) radiating outward from the center of rotation at increments of 2 pixels. All ROIs are nonoverlapping and the number of ROIs is unique to the size and radial length of the MF at a given level. The mean FI percent of each radial ROI in a single MF was calculated and plotted with a 3-point moving average, creating a fat distribution curve at every level, depicting the change in MF FI% moving radially (deep to superficial) through the muscle and away from the center of rotation. Each curve was distance normalized so that the x-axis ranged from 0% (deepest point) to 100% (most superficial point) of the radial muscle width. For repeatability, fat-maps were created at 2 axial slices at each disc level for each right and left MF. We then averaged the fat-map curves across the 2 axial slices, then across the right and left MF, resulting in a single MF fat-map at each lumbar level. Fat-maps were created using Python (Python Software Foundation, www.python.org).

### Statistical parametric mapping

We used statistical parametric mapping (SPM) to identify spatial patterns in the MF fat distribution and tested for associations with age, sex, BMI, disc degeneration, and pain. SPM is a “spatially extended statistical process” [21] that has been used to identify regional intramuscular damage accumulation in the quadriceps femoris [22]. For age and BMI, we used SPM canonical correlation analysis to identify age- and BMI-related patterns of FI across lumbar levels. Level-by-level posthoc analysis was conducted using SPM regression tests. For sex, we used the SPM Hotellings test to identify spatial patterns across levels. Level-by-level posthoc analysis was conducted using SPM unpaired t-tests. For pain and disc degeneration, we conducted level-by-level multivariable regression tests using SPM general linear models. For each analysis, we calculated the relevant SPM test statistic (SPM{X2}, SPM{T2}, SPM{t}) at every point from 0% to 100% of the fat-maps. Then, we calculated the critical threshold test statistic (*α*=0.05) so that any region of the fat-map where the SPM test statistic surpassed the critical threshold was deemed significant for a given analysis. The size of these significant regions (clusters) correlates with the strength of significance. All SPM analysis was performed using the SPM1D package in Python, which uses random field theory to control for multiple comparisons in SPM.

### Linear mixed effects modeling of FI

**Fig. 2.**
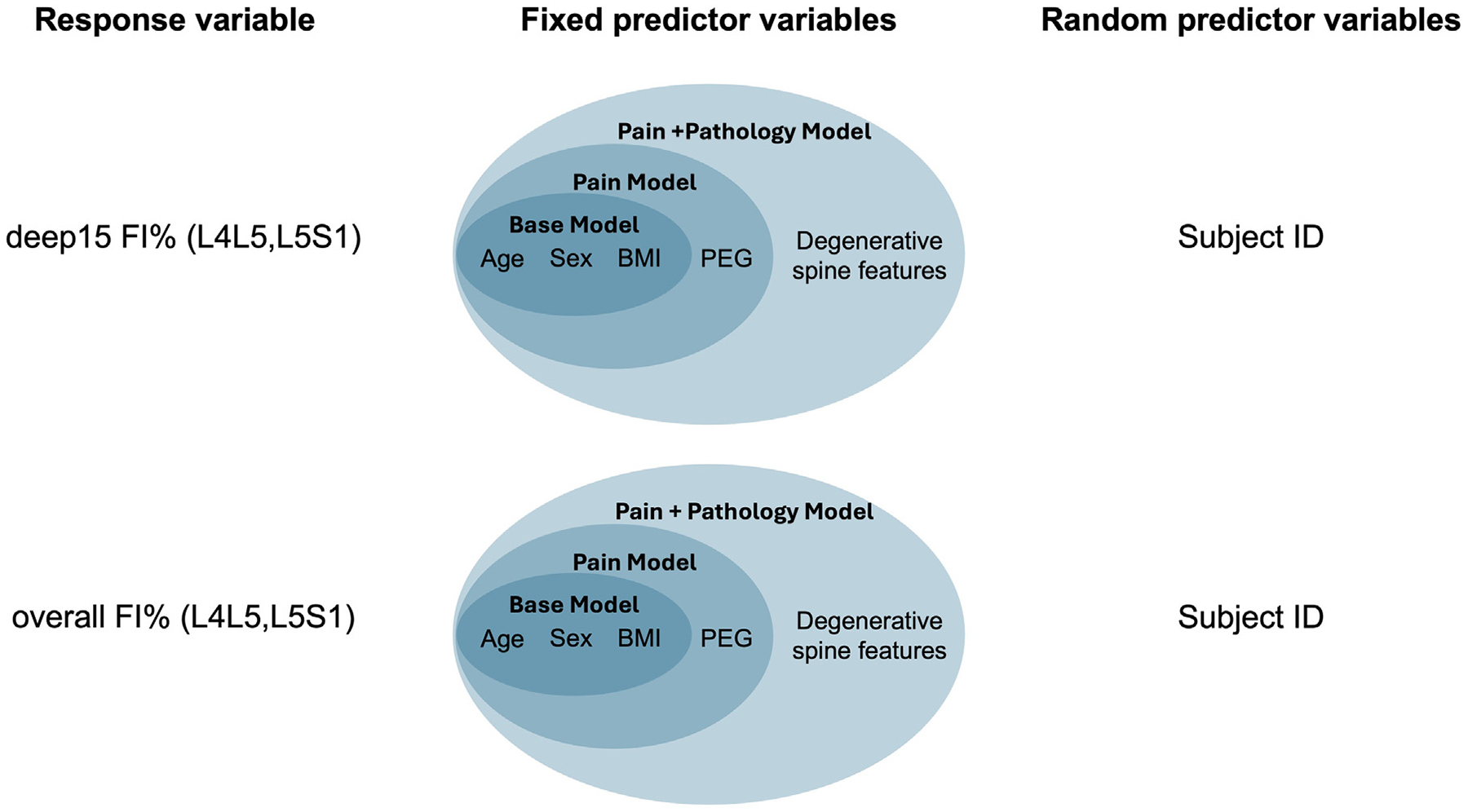
Model descriptions for linear mixed effects modeling. For both deep15 FI% and overall FI%, we created 3 nested linear mixed effects models, resulting in a total of 6 models.

Using the spatial analysis results, we identified a region of interest (ROI) within the MF, the deepest 15% of the MF at L4L5 and L5S1, where elevated FI was uniquely associated with disc degeneration and PEG score but not with BMI. We calculated the mean FI% within this ROI (deep15 FI%) in addition to the mean whole-muscle FI% (overall mean FI%) at both L4L5 and at L5S1. Both FI measures were separately compared to demographics, PEG, and adjacent degenerative spine features using a series of 3 nested linear mixed effects models (Fig. 2). For both overall mean FI% and deep15 FI%, we compared 3 nested models with the following fixed predictors:

1. Base Model: Age, sex, BMI, lumbar level
2. Pain Model: Base Model predictors + PEG
3. Pain + Pathology Model: Pain Model predictors + Adjacent degenerative spine features

Since these models included 2 measurements from each subject (one measurement at both L4L5 and L5S1), we included subject ID as a random effect in each model. Nested models were compared using ANOVA testing comparing log likelihood values. We then calculated conditional and marginal R-squared values to compare explained variance in the overall mean FI% models to the deep15 FI% models. All mixed effects modeling was conducted using the lme4 and lmerTest packages in R.

### Linear regression modeling of pain

Finally, we created 2 linear regression models with PEG as the response variable to compare deep15 FI% and overall mean FI% at the lower lumbar levels as predictors for pain. Each model used PEG as the outcome, but with different predictors:

1. Deep15 FI% model of PEG: mean deep15 FI%, age, sex, BMI, mean disc degeneration
2. Overall FI% model of PEG: mean overall FI%, age, sex, BMI, mean disc degeneration

These models were conducted using the mean disc degeneration and mean FI% values across L4L5 and L5S1.

## Results

### Demographics, degenerative imaging findings, and level-wise differences in fat distribution

**Fig. 3.**
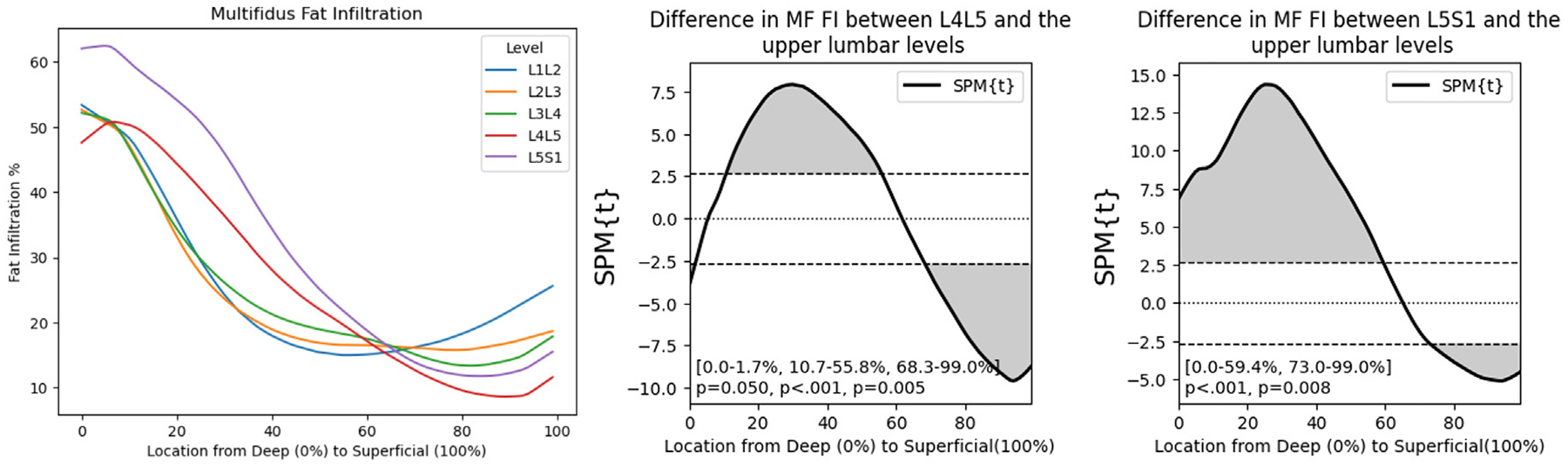
Multifidus fat distribution patterns differ across lumbar levels with lower lumbar levels having elevated fat infiltration in the deep region of the muscle. SPM t-tests showing regional differences in FI between upper lumbar levels and lower lumbar levels. Lower levels have higher FI in the deep MF and lower FI in the superficial MF compared to the upper lumbar levels.

Of the 230 patients, 106 were male and 124 were female with no significant differences in age or BMI between males (51.4 yrs, 26.0 kg/m^2^) and females (54.6 yrs, 26.0 kg/m^2^). From imaging, we calculated the incidence rate of each degenerative feature and the average overall FI% and deep15 FI% at each level (Table 1). There were no significant differences in overall FI% between the lumbar levels except at L5S1, where the overall FI% was on average 5.65% points higher than at any other lumbar level (Table 1). Using SPM paired t-tests, we find that lower lumbar levels (L4L5, L5S1) had elevated FI in the deepest 60% of the MF and lower levels of FI in the most superficial 40% of the MF compared to the upper lumbar levels (Fig. 3). There were no differences in MF fat distribution between right and left MF.

### Spatial distribution analysis: Age-, sex-, and BMI-related patterns

**Fig. 4.**
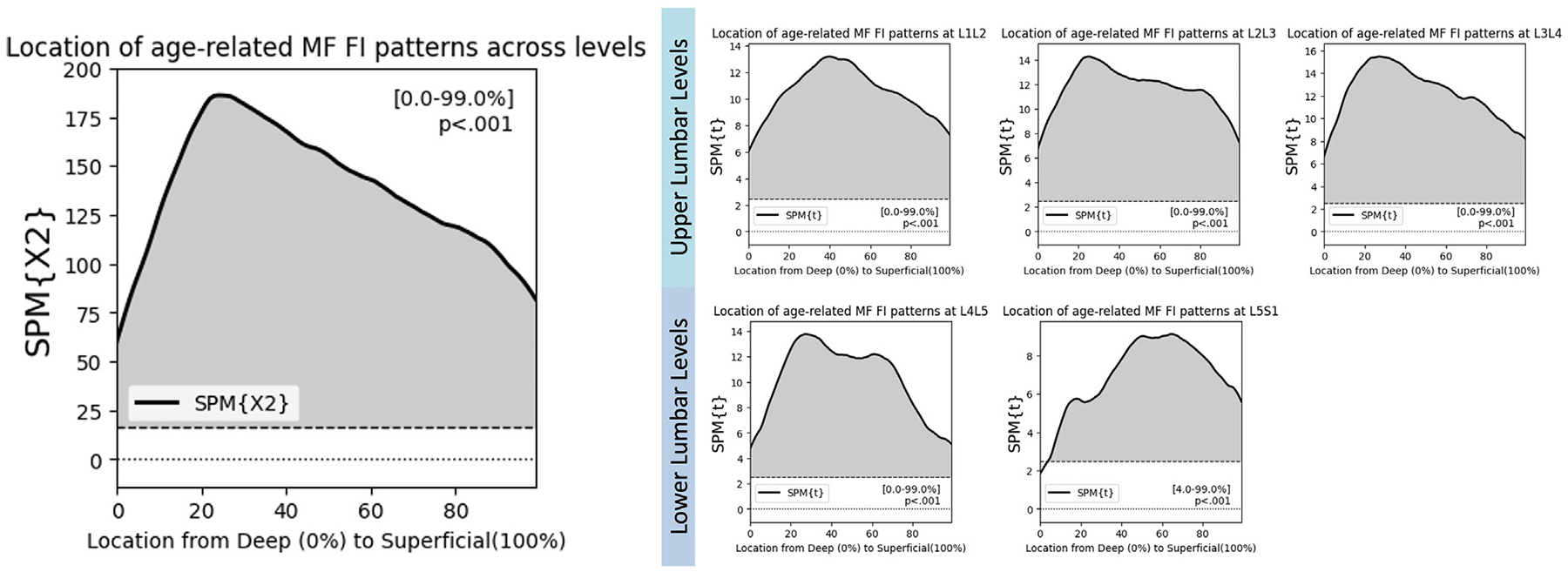
SPM Canonical correlation analysis shows that older age associates with elevated FI across the entire distribution of the multifidus. Posthoc testing confirms this relationship at every lumbar level.

**Fig. 5.**
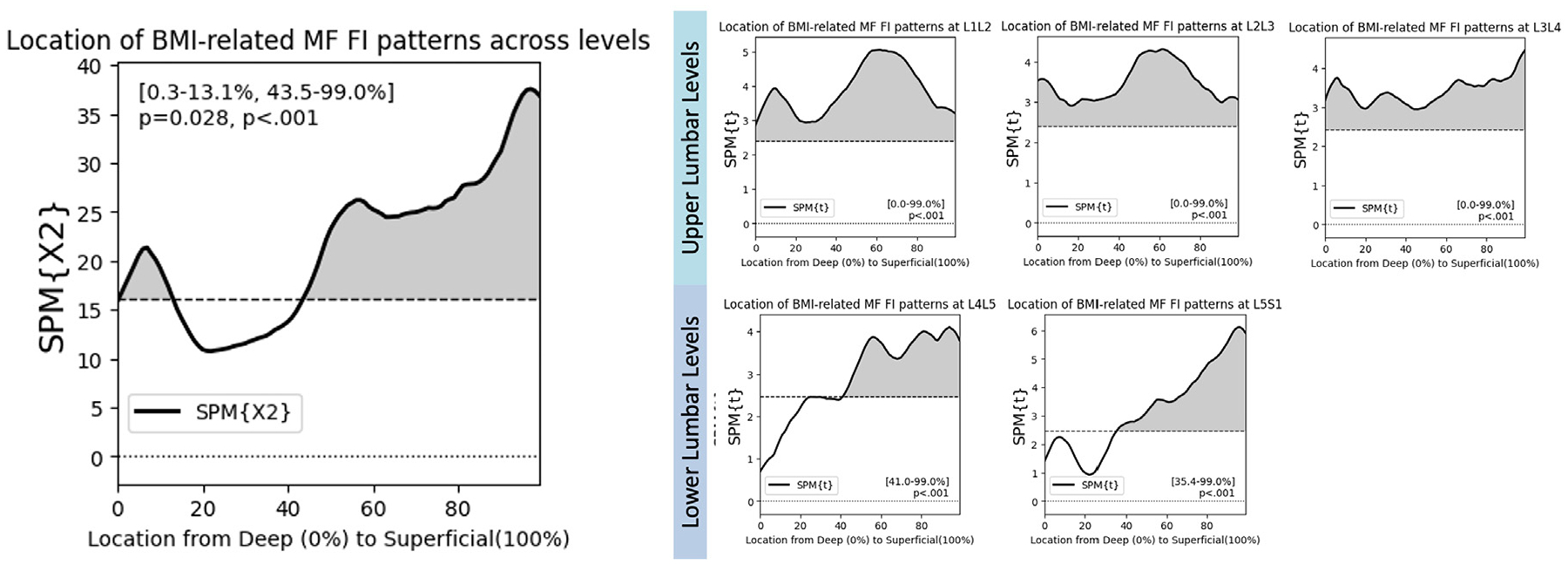
SPM Canonical correlation analysis shows that elevated BMI associates with higher levels of FI in different regions of the multifidus at the upper and lower levels. Posthoc testing reveals that this relationship is level specific. Interestingly, FI in the deep MF at L4L5 and L5S1 is not associated with BMI, but at upper lumbar levels, elevated BMI associates with elevated FI across the entire MF.

Using SPM canonical correlation analysis (CCA), we find that older age associated with elevated FI across the entire MF (Fig. 4, p<.001). Level-by-level posthoc analysis confirmed this association at each lumbar level (p<.001) except at L5S1, where age-related patterns of FI were observed across the entire distribution except for the deepest 5% of the MF (Fig. 4, p<.001). For BMI, we find an association between elevated BMI and elevated FI in 2 regions of the MF: the deepest 20% of the muscle (p=.02) and the most superficial 60% of the muscle (Fig. 5, p<.001). In a level-by-level posthoc analysis, elevated BMI associated with elevated FI across the entire distribution of the muscle at the upper 3 levels (Fig. 5, p<.001), but only in the most superficial 60% of the muscle at L4L5 and L5S1 (Fig. 5, p<.001).

**Fig. 6.**
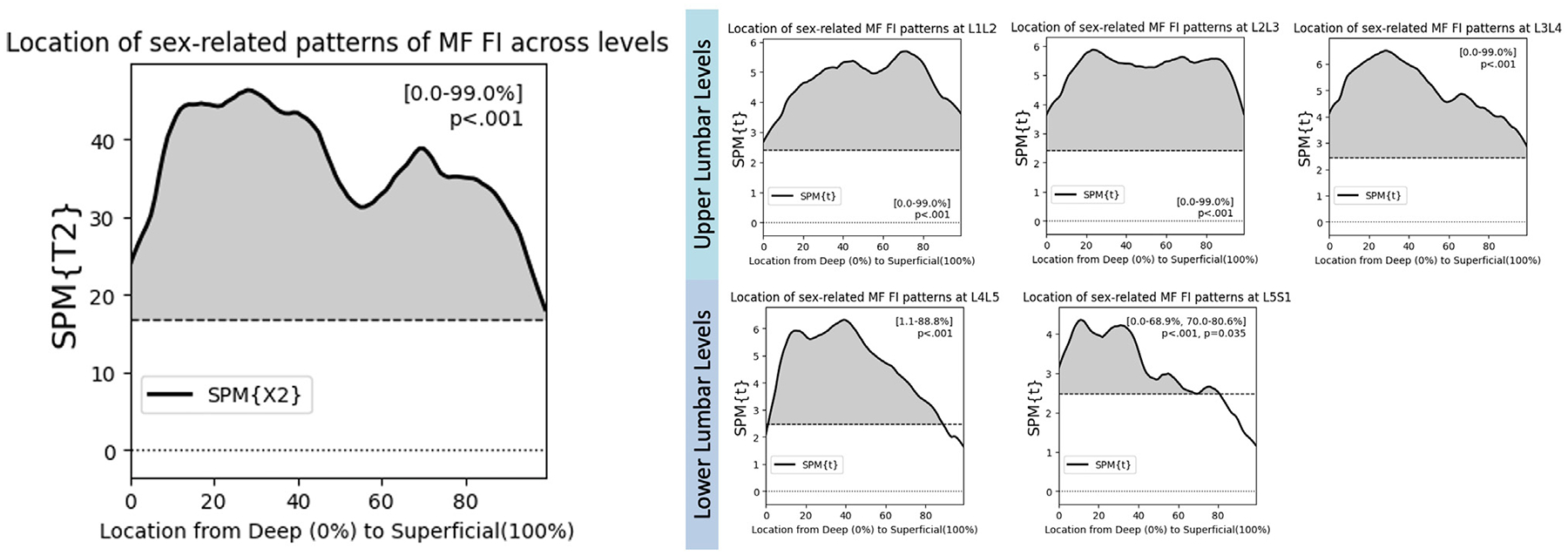
SPM Hotellings analysis shows that females have higher levels of FI across the entire distribution of the multifidus. Posthoc testing confirms this relationship at every lumbar level, except in the superficial MF at L4L5 and L5S1.

Using SPM Hotellings analysis, we find that females had higher levels of FI than males across the entire MF (Fig. 6, p<.001). In level-by-level posthoc testing, this association was observed at each upper level (Fig. 6, p<.001), but not at the lower lumbar levels, where there was no relationship between sex and FI in the most superficial 10% and the most superficial 20% of the MF at L4L5 and L5S1 respectively (Fig. 6, p<.001).

Overall, elevated MF FI at upper lumbar levels associated with older age, female sex, and higher BMI throughout the whole muscle. At lower lumbar levels, only older age and female sex associated with elevated FI across the whole muscle, whereas higher BMI only associated with MF FI in the superficial regions of the MF.

### Spatial distribution analysis: Disc degeneration- and pain-related patterns of FI

**Fig. 7.**
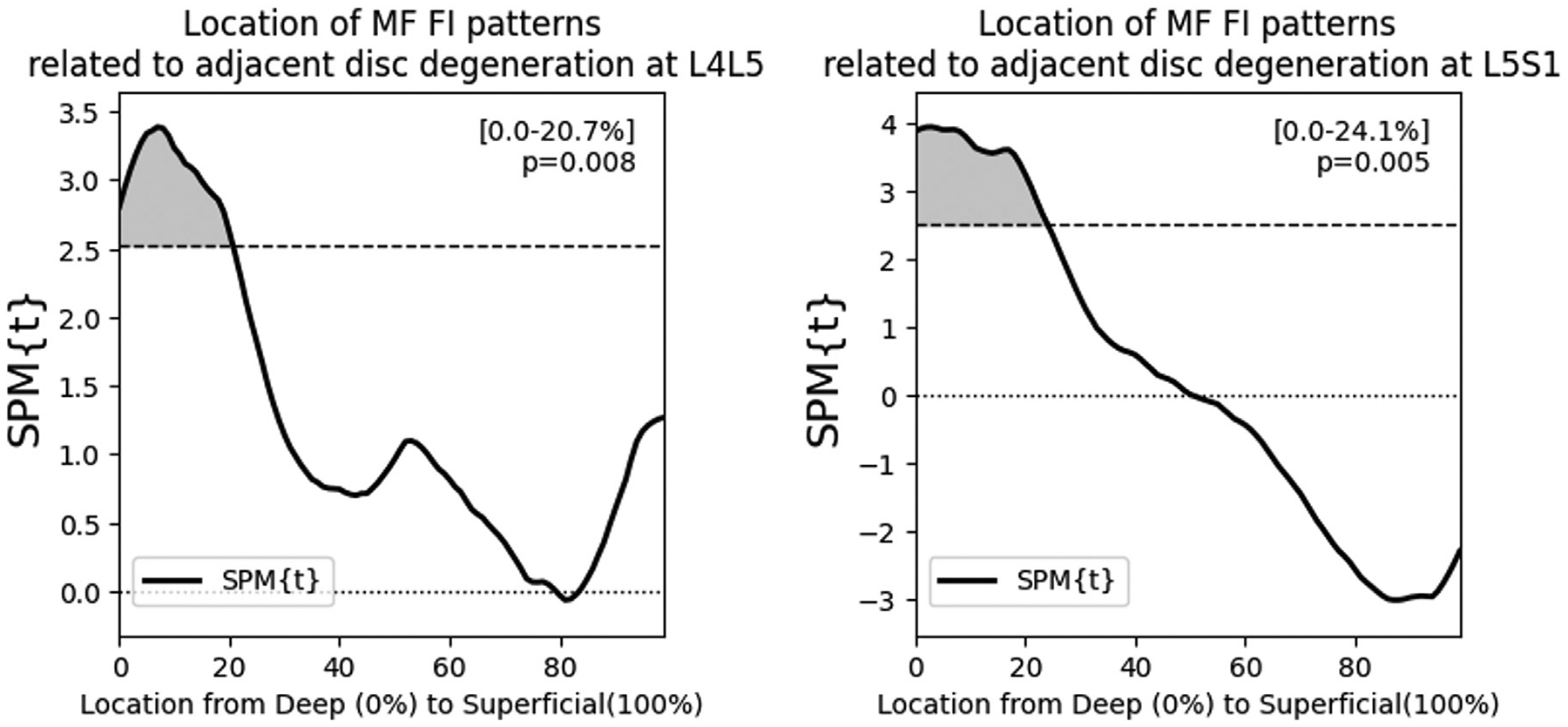
Multivariable regression accounting for age, sex, and BMI, identifies spatial patterns of FI associated with elevated adjacent disc degeneration. At lower levels, these disc degeneration related FI patterns are in the deep MF. At upper levels, an association is observed only at L1L2 in the superficial MF.

**Fig. 8.**
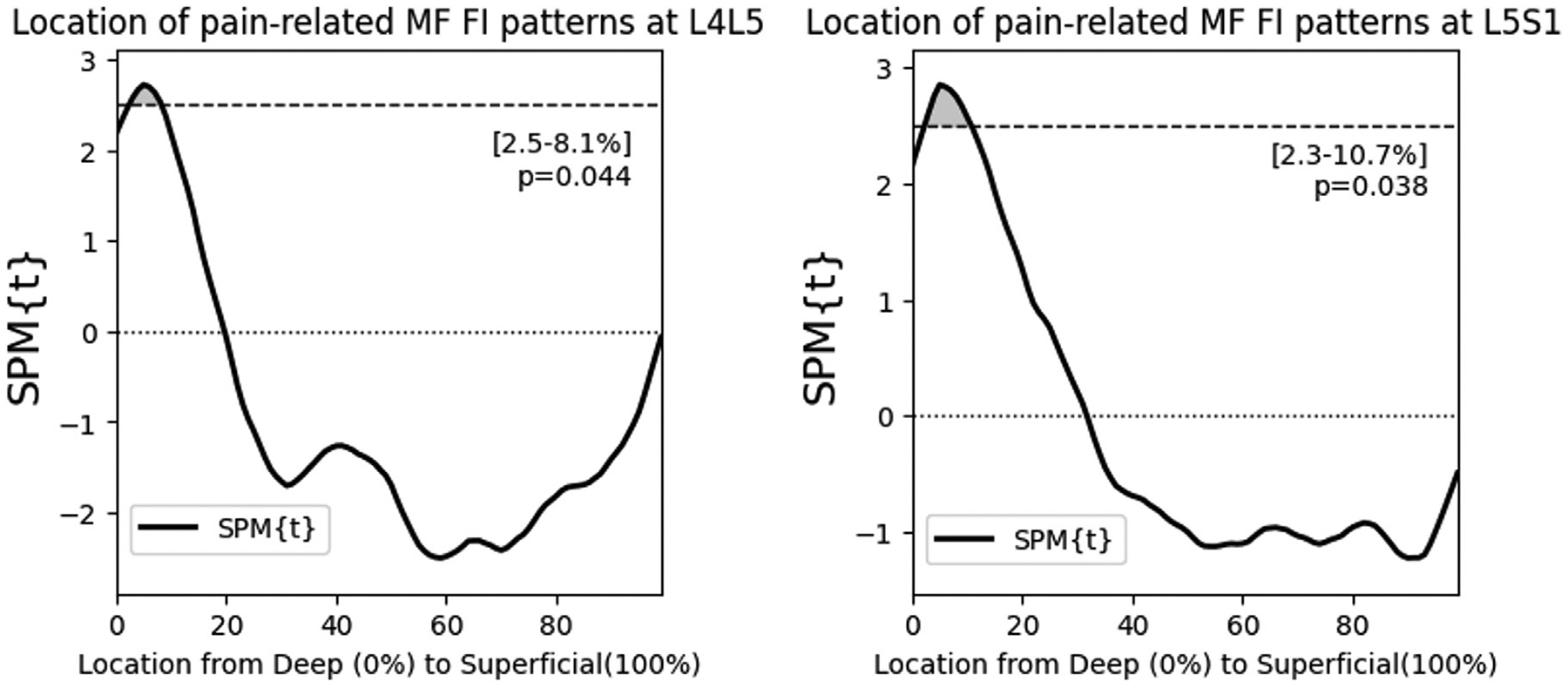
Multivariable regression accounting for age, sex, and BMI, shows that higher PEG score associates with distinct patterns of FI accumulation specifically in the deep MF at the lower lumbar levels. At upper levels, we did not find an association between PEG and FI distribution.

Using SPM general linear modeling, we find that higher levels of adjacent disc degeneration associated with elevated FI in the deep MF at the lower lumbar levels (L4L5, L5S1), independent of age, sex, and BMI (Fig. 7). This association was specific to the deepest 21% and the deepest 24% of the MF at L4L5 and L5S1 respectively (p<.01). In the upper lumbar levels, higher levels of adjacent disc degeneration associated with elevated FI only in the most superficial 40% of the MF at L1L2. At L2L3 and L3L4, disc degeneration was not associated with MF FI. For pain, we find that higher PEG scores associated with elevated FI at both L4L5 and L5S1, approximately within the deepest 10% of the MF (Fig. 8, p<.05). At the upper lumbar levels, PEG score did not associate with MF FI.

### Identification of a regionally specific standalone FI estimate for cLBP

**Fig. 9.**
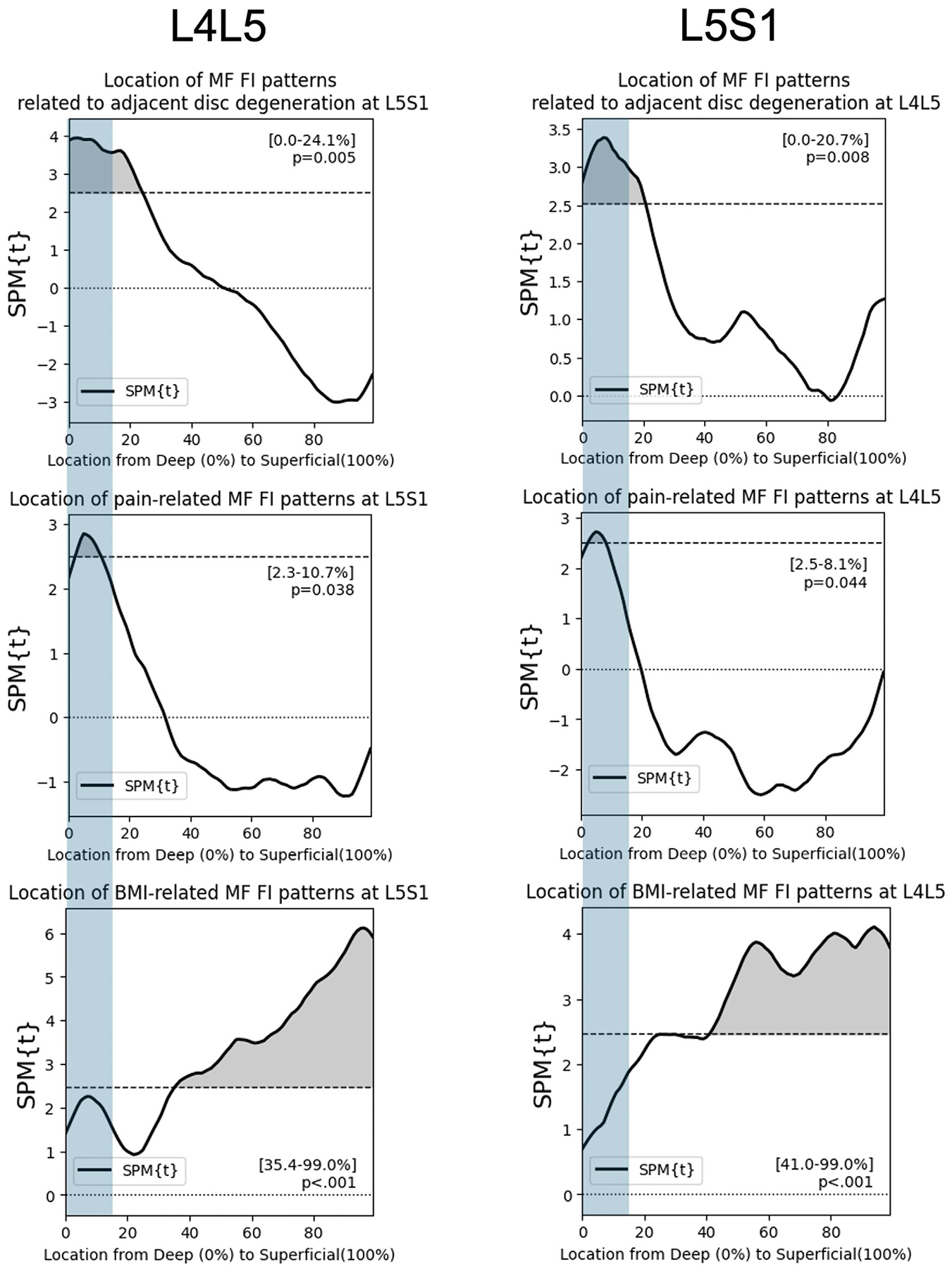
Elevated fat infiltration in the deepest 15% of the multifidus at L4L5 and L5S1 is uniquely associated with pain and disc degeneration but not with BMI.

From the above spatial analysis, we identified a region of the MF at the lower lumbar levels, the deepest 15% of the MF, that uniquely associated with pain and adjacent disc degeneration but not with BMI (Fig. 9). To further explore this relationship, we calculated the FI% of the deepest 15% of the MF (deep15 FI%) at L4L5 and at L5S1.

At L4L5 and at L5S1, the deep15 FI% was higher than the overall FI% by an average of 22.1% points and 27.6% points respectively. The deep15 FI% at L5S1 was higher than at L4L5 by an average of 10.2% points (Table 1).

From the first set of linear mixed effects model, the base models with age, sex, BMI, and level as fixed predictors, we find that older age and female sex associated with elevated deep15 FI% and with elevated overall FI%. Higher BMI did not associate with deep15 FI% but associated with elevated overall FI%. Further, age, sex, BMI, and level accounted for 43.7% of the variance in overall mean FI% but only 29.3% of the variance in deep15 FI% at L4L5 and L5S1.

From the pain models of deep15 FI% and overall FI%, which included PEG in addition to the base model, we find that higher PEG scores associated with elevated FI in the deep 15% of the MF, but not with elevated overall FI%. Using ANOVA and log-likelihood, we find that including PEG in the Pain Model yielded a significant improvement in model fit from the Base Model of age, sex, and BMI (p=.03). However, for the overall mean FI% models, the Pain Model did not improve model fit from the Base Model.

### Evaluating FI patterns linked to adjacent spine degenerative spine features

The last set of models, the Pain + Pathology models, included adjacent degenerative spine features in addition to the Pain Model. Adjacent disc degeneration was the only degenerative feature that associated with FI, with higher levels of disc degeneration associating with both elevated deep15 FI% and elevated overall mean FI%. No other degenerative feature associated with either measure of MF FI at the lower lumbar levels.

Using ANOVA and log-likelihood, we find that the inclusion of adjacent degenerative spine features in the Pain + Pathology model of deep15 FI% yielded a significant improvement in model fit from the Pain Model (p=.01). However, in the overall mean FI% modeling, degenerative spine features did not improve model fit.

Overall, the inclusion of PEG score and adjacent degenerative spine features improved our modeling of deep15 FI% beyond the Base Model of age, sex, BMI, and level. However, for overall FI%, PEG score and adjacent degenerative spine features did not improve model fit beyond the Base Model. All model estimates and p-values for all variables, and marginal r-squared values for all models are reported in Tables 2 and 3.

### Evaluating summary measures and region-specific measures of FI as predictors of pain

From linear regression modeling of PEG as the response variable, we find that higher mean deep15 FI% at the lower lumbar levels associated with higher PEG score (p=.023). Mean overall FI% did not associate with PEG score. Further, neither mean disc degeneration nor age or sex associated with PEG in either model, but higher BMI associated with higher PEG scores in both models (p<.05). Overall, the mean overall FI% of the MF at L4L5 and L5S1 did not associate with PEG, but elevated FI specifically in the deepest 15% of the muscle associated with higher PEG scores.

## Discussion

Using a novel approach to fat-map the MF, we find that pain and adjacent disc degeneration associate with elevated FI specifically in deep regions of the MF at the lower lumbar levels, independent of age, sex, and BMI. Further, we use linear mixed effects modeling to show that fat fraction in the deepest 15% of the MF, the deep15 FI%, at lower lumbar levels is more strongly associated with cLBP symptoms and degenerative features and less associated with age, sex, and BMI than overall FI%. Spatial parametric mapping of the MF fat distribution at L4L5 and L5S1 show that fat content in the deep regions of the MF closest to the vertebral center of rotation is uniquely associated with higher PEG scores and higher levels of adjacent disc degeneration, but not with BMI (Fig. 9), highlighting a pattern that warrants further investigation. This finding is corroborated by the linear mixed effects modeling results, which show that the deep15 FI% of the MF is associated with both PEG score and adjacent disc degeneration. While overall FI% is also associated with adjacent disc degeneration, this association does not improve model fit from the Base Model of age, sex, BMI, and Level. Further, age, sex, BMI, and level explain a larger proportion of the variance in overall FI% (43.7%) than in deep15 FI% (29.3%). In linear regression models of PEG, we find that only the mean deep15 FI% and not the mean overall FI% at the lower lumbar levels is associated with higher levels of pain and pain interference in cLBP patients, supporting its potential as a feature of interest for future prospective study.

There are several structural and functional differences between deep and superficial fascicles of the MF that may explain regional differences in FI and the specificity of the FI to cLBP. First, we find that these regional differences are, in part, correlated to the degeneration of the adjacent disc. We find that the fibers of the MF closest to the disc are both more fatty infiltrated and more associated with adjacent disc degeneration than more superficial regions of the muscle. In recent study of MF FI in the coronal plane, the most fatty-infiltrated region of the muscle at each vertebral level was at the spinous process and lamina [14]. This pinpoints the FI accumulation to the attachment sites of the deep fascicles of the MF. Due to its location and strict function as a local stabilizer, the deep fascicles of the MF may be more vulnerable to disuse with the loss of disc height and compliance at the adjacent disc, lending the muscle more susceptible to FI.

Further, we find that higher PEG scores, a comprehensive measure of pain and pain interference, associate with elevated FI in the deep MF at L4L5 and L5S1. The mechanisms underlying this association are unclear, but the loss of stability driven by the degeneration of both the intervertebral disc and the deep fascicles of the MF is thought to put the adjacent osseoligamentous tissues under higher loading and at higher risk of injury. However, through reflex inhibition, this relationship is bidirectional, as several studies have shown changes in neural drive and MF recruitment with pain [23,24] which may contribute to MF degeneration. The deep and superficial fascicles of the MF are differentially active [25], suggesting that reflex inhibition could have differential effects within the MF. This study provides regional context for the association between cLBP symptoms and MF degeneration that suggest that structural and functional differences between the deep and superficial regions of the MF may influence patterns of MF degeneration in cLBP. Further study is necessary to determine the underlying causal mechanisms linking pain to MF degeneration.

While cLBP symptoms and adjacent disc degeneration associate with FI patterns in the deep region of the MF at L4L5 and L5S1, we find that this association is specific to the lower lumbar levels. Due to the curvature of the lumbar spine and the orientation of the MF fascicles, there are important biomechanical differences between the upper and lower lumbar levels [9]. The lower lumbar levels are of particular interest in cLBP as they experience higher loads and typically degenerate faster than upper lumbar levels. In addition, the health of the adjacent disc at lower lumbar levels may be more relevant to cLBP than at the upper lumbar levels [26]. We find that this may also be true for MF quality. Unlike MF FI at the lower lumbar levels, MF FI at the upper lumbar levels did not associate with both pain and disc degeneration in any region of the muscle, but did associate with age, sex, and BMI across the entire MF.

The difference in findings between upper and lower lumbar levels suggest that MF quality at lower lumbar levels may be more strongly associated with cLBP than at upper levels. Further, the mechanisms of FI in the MF may differ between levels. Biomechanically, the lower lumbar levels are most affected by changes in lumbar lordosis. While we did not measure differences in lordosis, segmental instability and loss of lordosis associated with the concurrent degeneration of the disc and the deep fascicles of the MF may be more likely to cause tissue damage and pain in lower lumbar levels experiencing higher tissue loads. In turn, this may render the deep MF more susceptible to degeneration, as segmental stability may be more compromised by adjacent disc degeneration at the lower lumbar levels than at upper levels.

Surprisingly, beyond adjacent disc degeneration, our results do not show an association between any other degenerative spine feature and MF FI at the lower lumbar levels. Prior work has identified a relationship between MF FI and adjacent cartilage endplate damage [18], Modic change [27], and facet joint osteoarthritis [28]. However, this relationship is not supported by the results of this study which accounts for age, sex, and BMI and considers multiple spine features in a single analysis. Interestingly, we do not find a relationship between adjacent nerve root contact or central canal stenosis and MF FI patterns. However, neither the presence of nerve root contact nor central canal stenosis explicitly suggest denervation of the MF. In animal models, rapid muscle degeneration has been reported in the MF immediately following an induced injury to the disc [29]. Still, the lasting effects of FI in the muscle are unclear and the degree to which fatty infiltrated muscle may be regenerated is not well understood. Although our results suggest that, except for adjacent disc degeneration, the degenerative spine features tested do not associate with MF FI, future work is necessary to investigate the time-scale of changes in MF FI in cLBP patients following the development or progression of degenerative features in the passive tissues.

Prior work has shown that the MF appears to have different quantities of FI regionally [30]. Our findings show that in the context of cLBP, FI in the deep regions of the MF appears to be more strongly associated with cLBP symptoms and disc degeneration than in other regions of the muscle. Although the role and underlying mechanisms of MF degeneration in cLBP is unclear, most prior work cited in literature reviews [31,32] rely on summary measures of FI agnostic of location within the MF. Underlying mechanisms of FI may have differential effects on the MF that are regionally dependent on structural and functional differences between deep and superficial fascicles of the muscle. Our findings suggest that overall FI% may be less associated with cLBP symptoms and degenerative features than the deep15 FI%. Further, while our results show that age and sex associate with FI patterns across the entire length of the MF, we find that age and sex explain less of the variance in FI in the deep regions of the MF than in the overall muscle. Regionally-informed reporting of MF FI may improve our understanding of the role of MF degeneration in cLBP and provide important regional context for further exploration of causal mechanisms.

Strengths of this study include a novel spatial approach for quantifying muscle FI, and our utilization of an advanced MRI approach with substantial implications for studying degenerative changes in spinal muscles [33]. However, there are several limitations that are important for the interpretation of our findings. We measure the distribution of FI in only one direction, moving radially from the vertebral center of rotation. As there are no distinct cleavage planes between deep and superficial fascicles of the MF, we are only able to identify deep and superficial regions of the MF relative to the vertebral center of rotation. In addition, while we provide important regional context, further study with longitudinal data or using animal models are necessary to directly test causal mechanisms.

Our findings have potential clinical implications for cLBP. First, the overall FI% of the MF, the current standard for reporting MF quality in the literature, appears to be less associated with cLBP symptoms and degenerative spine features and more explained by age, sex, and BMI than the FI% of the deep MF. The FI% of the deep region of the MF appears to be a muscle quality biomarker that may be relevant for cLBP, although further validation in longitudinal or interventional study is necessary. It could also be a target for intervention, but there is limited evidence that FI may be slowed, and it is unclear if skeletal muscle FI is reversible. Some studies suggest that physical activity regiments may slow age-related FI in skeletal muscle broadly, but the relevance of this finding to cLBP is not well understood [34]. Future studies are necessary to establish the efficacy of physical activity interventions in slowing FI in cLBP patients and determine if specific regiments could target the deep regions of the MF. Lastly, although FI in the deep region of the MF at the lower lumbar levels appears to be more strongly associated with cLBP symptoms and degenerative spine features, the overall FI% may still hold important clinical relevance in cLBP.

In summary, these results show that fat content in the deep regions of the MF at the lower lumbar levels is more sensitive to cLBP symptoms and adjacent disc degeneration than overall FI%, while the overall FI% appears to be more associated with age, sex, and BMI. Overall, these findings highlight regional differences in cLBP-related FI within the MF which improves our understanding of cLBP-related MF degeneration and provides additional context for possible causal mechanisms of FI. Further, this presents a novel measure of MF FI specific to the deep MF, the deep15 FI%, that is more strongly associated with cLBP than summary measures, which may have potential clinical implications.

## Figures and Tables

**Table 1:** Incidence of degenerative spine features, mean disc degeneration, and mean FI values across lumbar levels

	L1L2	L2L3	L3L4	L4L5	L5S1
Modic change	30 (13%)	49 (21.3%)	55 (23.9%)	73 (31.7%)	86 (37.4%)
Endplate defect	49 (21.3%)	48 (20.9%)	49 (21.3%)	73 (31.7%)	84 (36.5%)
Disc degeneration	2.76±1	2.96±1.1	3.1±1.1	3.42±1.1	3.4±1.1
Nerve root contact	11 (4.8%)	37 (16.1%)	70 (30.4%)	119 (51.7%)	81 (35.2%)
Facet joint osteoarthritis	56 (24.3%)	77 (33.5%)	134 (58.3%)	182 (79.1%)	172 (74.8%)
Central canal stenosis	16 (7%)	47 (20.4%)	87 (37.8%)	111 (48.3%)	39 (17%)
overall FI%	25.06±10.2	24.6±11.4	24.46±12.1	25.55±11.4	30.57±12
deep15 FI%	42.21±17.6	41.23±15.8	41.88±15.3	47.65±13.9	58.19±15.5

**Table 2:** Model estimates, confidence intervals (CI) calculated using likelihood profiles, and p-values for all fixed effects across all models from the lower lumbar levels

Base model:								
	Deep15%, lower lumbar levels				Overall FI%, lower lumbar levels			
Fixed effect	Estimate	2.5% CI	97.5% CI	p-val	Estimate	2.5% CI	97.5% CI	p-val
Intercept	46.40	44.09	48.71	p<.001	22.22	20.60	23.85	p<.001
AGE	4.77	3.32	6.22	p<.001	6.23	5.16	7.29	p<.001
SEX: F	7.16	4.28	10.04	p<.001	6.16	4.05	8.27	p<.001
BMI	0.80	−0.65	2.24	0.284	1.36	0.30	2.42	0.013
Level: L5S1	11.32	9.43	13.22	p<.001	5.02	4.02	6.02	p<.001
Pain model:								
	Deep15%, lower lumbar levels				Overall FI%, lower lumbar levels			
Fixed effect	Estimate	2.5% CI	97.5% CI	p-val	Estimate	2.5% CI	97.5% CI	p-val
Intercept	46.45	44.16	48.74	p<.001	22.22	20.59	23.85	p<.001
PEG	1.58	0.15	3.01	0.033	−0.35	−1.40	0.71	0.524
AGE	4.87	3.43	6.31	p<.001	6.21	5.14	7.27	p<.001
SEX: F	7.08	4.23	9.93	p<.001	6.18	4.07	8.29	p<.001
BMI	0.57	−0.88	2.02	0.443	1.41	0.34	2.48	0.011
Level: L5S1	11.32	9.43	13.22	p<.001	5.02	4.02	6.02	p<.001
Pain + Pathology model:								
	Deep15%, lower lumbar levels				Overall FI%, lower lumbar levels			
Fixed effect	Estimate	2.5% CI	97.5% CI	p-val	Estimate	2.5% CI	97.5% CI	p-val
Intercept	47.13	43.54	9.17	p<.001	22.59	20.25	24.93	p<.001
PEG	1.65	0.26	3.04	0.022	−0.35	−1.41	0.70	0.519
DDD	2.51	0.81	4.22	0.005	1.32	0.28	2.37	0.015
MC	0.02	−3.54	3.52	0.990	−0.93	−2.97	1.10	0.375
EP	0.20	−3.53	3.97	0.918	−1.09	−3.28	1.13	0.339
FJOA	−0.35	−3.32	2.64	0.818	−0.44	−2.24	1.36	0.638
NRIN	0.45	−2.26	3.17	0.747	0.87	−0.73	2.46	0.290
CCS	−2.53	−5.66	0.55	0.112	−0.15	−2.00	1.68	0.871
AGE	4.33	2.83	5.83	p<.001	5.98	4.87	7.09	p<.001
SEX: F	6.82	4.03	9.61	p<.001	5.98	3.87	8.09	p<.001
BMI	0.56	−0.85	1.97	0.441	1.41	0.34	2.48	0.011
Level: L5S1	10.63	8.52	12.71	p<.001	5.23	4.09	6.35	p<.001

PEG, pain, enjoyment of life, and general activity survey score; DDD, adjacent disc degeneration; MC, Modic changes; EP, endplate defects; FJOA, facet joint osteoarthritis; NRIN, nerve root contact; CCS, central canal stenosis.

PEG, disc degeneration, age, and sex are significantly associated with deep15 FI% across models, while disc degeneration, age, sex, and BMI are significantly associated with overall FI%.

**Table 3:** Marginal r-squared for all models

	Deep15%, lower lumbar levels	Overall FI%, lower lumbar levels
Model	Marginal r-squared	Marginal r-squared
Base Model	0.293	0.437
Pain Model	0.302	0.437
Pain +Pathology Model	0.325	0.442

Marginal r-squared values across models show that the Base Model of age, sex, BMI, and level explains more of the variance in overall FI% than in deep15 FI%.
